# Is Weight Loss the Main Driver for A1C Improvement by Glucagon‐Like Peptide 1 (GLP‐1) Receptor Agonists? A 2.5‐Year Analysis in Real‐World Clinical Practice

**DOI:** 10.1111/1753-0407.70054

**Published:** 2025-01-23

**Authors:** Marwa Al‐Badri, Shilton Dhaver, Osama Hamdy

**Affiliations:** ^1^ Joslin Diabetes Center, Affiliated With Harvard Medical School Boston Massachusetts USA; ^2^ Harvard Medical School Boston Massachusetts USA

**Keywords:** GLP‐1 receptor agonists, glycemic control, real‐world clinical practice, type 2 diabetes, weight loss

## Abstract

**Background:**

Glucagon‐like peptide‐1 receptor agonists (GLP‐1 RAs) are established treatment options for type 2 diabetes (T2D). In addition to their glycemic benefit, GLP‐1 RAs also induce weight loss by suppressing appetite via hypothalamic pathways. However, it remains unclear whether weight reduction is the primary driver of glycemic improvement.

**Methods:**

We retrospectively evaluated 256 patients with T2D who were treated with exenatide (*n* = 84), dulaglutide (*n* = 99), or semaglutide (*n* = 73) for 2.5 years without interruption in real‐world clinical practice. Body weight and A1C were measured every 6 months. Baseline characteristics included an average age of 61.8 ± 11.9 years, 51.5% female, diabetes duration of 12.9 ± 8.3 years, weight of 103.1 ± 20.7 kg, BMI of 35.7 ± 7.5 kg/m^2^, and A1C of 8.2% ± 1.5%. Patients were stratified into tertiles based on percentage weight change at 2.5 years within the overall cohort and for each GLP‐1 RA group.

**Results:**

The first tertile experienced an average weight loss of −12.2% ± 5.7% (*p* < 0.0001), the second tertile lost −3.5% ± 1.4% (*p* < 0.0001), and the third tertile gained +2.8% ± 3.4% (*p* < 0.0001). The average changes in A1C were − 0.98 ± 1.8% (*p* < 0.0001), −0.56% ± 1.4% (*p* < 0.001), and −0.19% ± 1.9% (*p* = 0.4), respectively. A1C strongly correlated with weight change (*p* < 0.001). The same observations were reproducible in each medication group.

**Conclusions:**

These findings suggest that the long‐term improvement in glycemic control associated with GLP‐1 RA therapy is primarily driven by weight loss rather than any other intrinsic effect of GLP‐1 RA. This highlights the importance of weight reduction as a key therapeutic target for optimizing glycemic outcomes in patients with T2D receiving GLP‐1 RAs.

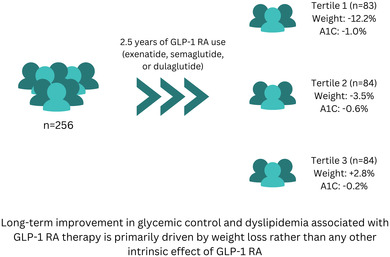

## Graphical abstract

This retrospective study of 256 patients reveals a strong correlation between percentage weight change and A1C reduction in patients on long‐term GLP‐1 RA use. Additionally, patients who lost more weight had significant improvements in dyslipidemia. These findings emphasize weight reduction as a crucial therapeutic target to enhance metabolic outcomes in real‐world clinical practice.

**Summary**

- Long‐term improvement in glycemic control and lipid profile with GLP‐1 RAs is primarily driven by weight loss, especially with long‐acting GLP‐1 RAs.
- Patients with minimal weight loss or weight gain during long‐term GLP‐1 RA use may not experience significant improvements in glycemic control.
- Future research should explore the long‐term effects of GLP‐1 RAs in more diverse populations to validate and extend these findings.

## 1 Introduction

Type 2 diabetes (T2D) is a metabolic disorder that is mainly characterized by hyperglycemia and insulin resistance. Glucagon‐like peptide‐1 receptor agonists (GLP‐1 RAs), also known as incretin mimetics, are a class of drugs that effectively improve glycemic control through different intrinsic effects like increasing insulin secretion, suppressing glucagon secretion, and slowing gastric emptying [1]. They also induce weight loss, by hypothalamic suppression of appetite [1]. Short‐acting agents such as exenatide, administered twice daily, showed diminishing effectiveness in controlling overnight and fasting plasma glucose, but sustained its impact on gastric emptying upon long‐term use [1, 2]. Conversely, longer‐acting GLP‐1 RAs, like liraglutide, once‐weekly exenatide, dulaglutide, albiglutide, and semaglutide have more pronounced long‐term effects on overnight and fasting plasma glucose and A1C [3, 4, 5]. This holds true both when used alongside oral glucose‐lowering agents or combined with basal insulin [1]. Recently, most of these drugs showed significant improvement in major adverse cardiovascular events (MACE) [3, 6, 7, 8]. Semaglutide was shown to slow the progression of renal impairment in patients with chronic kidney disease [9]. Consequently, guidelines specifically recommend GLP‐1 RA as the initial pharmacological intervention for patients with pre‐existing atherosclerotic cardiovascular disease and for patients with CKD if SGLT2‐inhibitors are not suitable [10]. It is well‐known that weight loss per se significantly improves insulin sensitivity and consequently improves A1C and other cardiovascular risk factors [11]. However, it is unclear whether the long‐term glycemic benefits of GLP‐1 RAs are primarily driven by weight reduction or other intrinsic effects on pancreatic cells.

In this study, we aim to investigate whether long‐term improvement in glycemic control in real‐world clinical practice is a result of weight loss or other intrinsic mechanisms of GLP‐1 RA.

## 2 Methods

### 2.1 Study Design and Participants

This is a retrospective analysis of electronic health records (EHR) of patients with T2D at Joslin Diabetes Center in Boston, MA, who were treated with GLP‐1 RAs (exenatide, dulaglutide, or semaglutide) continuously for 2.5 years without interruption.

We searched EHR for patients with T2D who started exenatide during the period between 2005 and 2006 and dulaglutide or semaglutide during the period between 2018 and 2019. Eligible patients were older than 18 years of age, had a diagnosis of T2D, continued GLP‐1 RA without interruption for 2.5 years, and had anthropometric and laboratory data available. Patients were excluded from the analysis if they stopped GLP‐1 RA within 2.5 years or had incomplete data after baseline measurements. We collected the following information: age, sex, diabetes duration, weight, body mass index (BMI), A1C, systolic blood pressure (SBP), diastolic blood pressure (DBP), triglycerides, LDL‐cholesterol, HDL‐cholesterol, ALT, AST, serum creatinine, eGFR, urinary microalbumin/creatinine ratio, number of anti‐hyperglycemic medications, number of anti‐hypertensive medications, and the insulin dose, if used. Baseline measurements were recorded at the start of GLP‐1 RA, and at approximately 6‐month intervals for 2.5 years.

### 2.2 Study Participants

The study was approved by the Committee on Human Studies (CHS) at Joslin Diabetes Center (ID# STUDY00000200). Out of the 156 patients who started exenatide between 2015 and 2016, only 84 patients (group A) met our selection criteria and out of the 820 patients who started either dulaglutide or semaglutide between 2018 and 2019, 99 patients on dulaglutide (group B) and 73 patients on semaglutide (group C) met our selection criteria. Thus, a total of 256 patients were included in this analysis. Average age was 61.8 ± 11.9 years, 51.5% female, diabetes duration 12.9 ± 8.3 years, weight 103.1 ± 20.7 kg, BMI 35.7 ± 7.5 kg/m^2^, and A1C 8.2% ± 1.5%. Detailed demographic data of the study population are shown in Table 1. The study cohort was classified collectively and in each medication group into tertiles according to the percentage of weight loss at 2.5 years.

**TABLE 1:** Baseline demographic and clinical characteristics of participants.

	All participants (*n* = 256)
Age (years)	61.8 ± 11.9
Female sex (%)	51.5
Duration of diabetes (years)	12.9 ± 8.3
Weight (kg)	103.1 ± 20.7
Body mass index (kg/m^2^)	35.7 ± 7.5
A1 C (%)	8.2 ± 1.5
Systolic blood pressure (mmHg)	129.2 ± 14.5
Diastolic blood pressure (mmHg)	76.2 ± 9.4
Total cholesterol (mg/dL)	159.2 ± 43.2
LDL‐cholesterol (mg/dL)	82.1 ± 32.9
HDL‐cholesterol (mg/dL)	47.8 ± 13.5
Triglycerides (mg/dL)	180.6 ± 103.9
ALT (U/L)	26.2 ± 15.1
AST (U/L)	22.8 ± 11.8
Serum creatinine (mg/dL)	0.9 ± 0.3
Urinary microalbumin/creatinine ratio (μg/mg)	92.3 ± 218.8
eGFR (mL/min/1.73 m^2^)	85.7 ± 27.4
C‐peptide (ng/mL)	4.7 ± 3.6
Number of diabetes medications	1.37 ± 0.9
Number of antihypertensive medications	1.37 ± 1.14
Short acting insulin (units)	18.7 ± 33.4
Long acting insulin (units)	25.7 ± 34.6

*Note:* Data are given as mean ± SD.

### 2.3 Statistical Analysis

Demographic and baseline characteristics were evaluated using descriptive statistics. Continuous variables are reported as mean ± standard deviation (SD) unless otherwise specified. Categorical variables are presented as percentages. Since clinic visits during the follow‐up period were not rigorously scheduled every 6 months, an approximation of each visit time to the nearest 6‐month timeline was used. If data were missing, the last observation was carried forward provided that two consecutive data points were not missed. Chi‐square test, Fisher exact test, and two‐sample *t*‐tests were used to compare baseline characteristics versus endpoints. Pairwise correlation was used to determine differences in A1C and weight over time for the whole cohort. All analyses were performed using STATA/SE version 17.0 for Windows (StataCorp, College Station, Texas, USA 2021). In all tests, *p* < 0.05 was considered statistically significant.

## 3 Results

**FIGURE 1:**
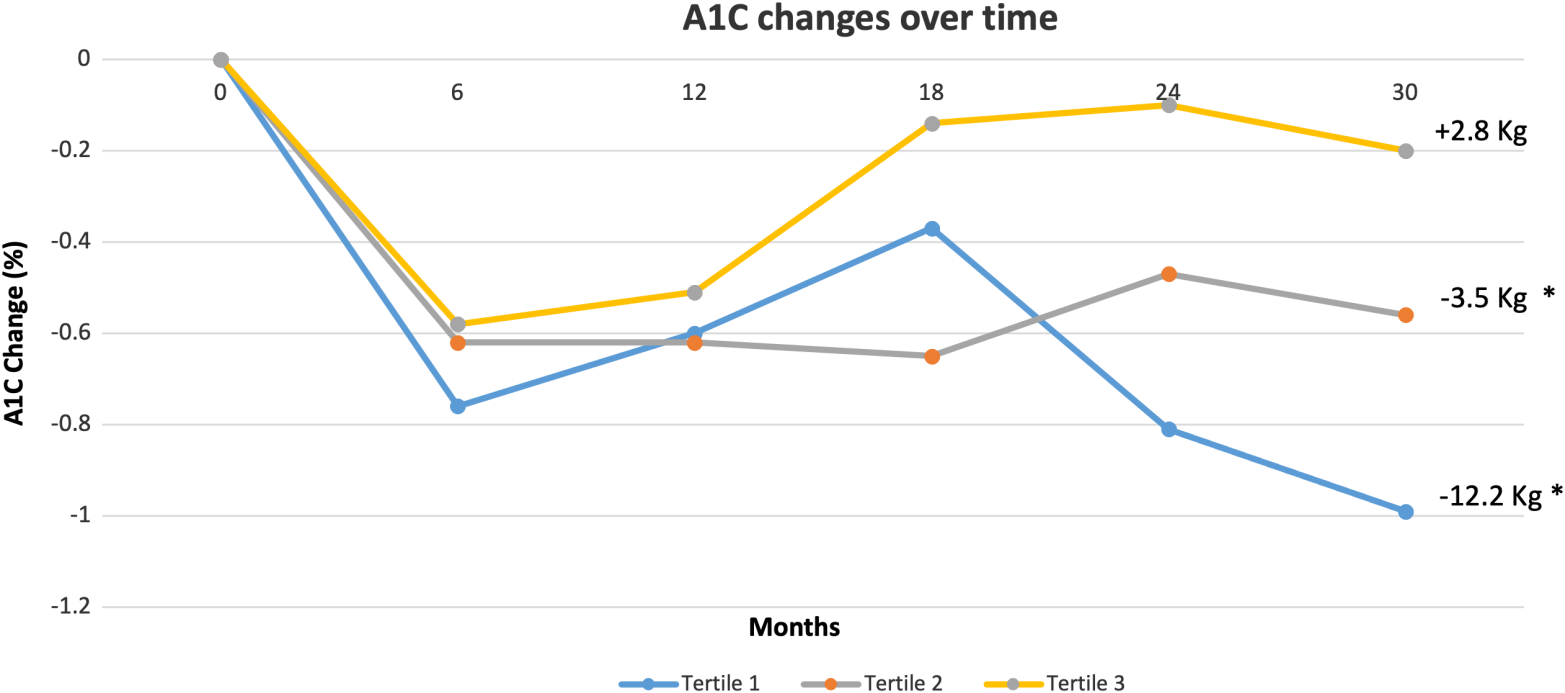
Change in A1C from baseline in each tertile of weight loss over 2.5 years among all participants. Tertile 1 (*n* = 83), Tertile 2 (*n* = 84), Tertile 3 (*n* = 84), Data are given as mean ± SD. *p < 0.05 compared to baseline.

We examined the relationship between the change in body weight and the change in A1C over time in the entire cohort (Figure 1). At 2.5 years, patients lost an average of 4.5 ± 7.8 kg, which corresponds to a weight loss of 4.3% ± 7.3% of the initial body weight (*p* < 0.0001 from baseline), and A1C decreased by 0.6% ± 1.7%, (*p* < 0.0001) compared to baseline (Figure 1). By using pairwise comparison, we found that A1C strongly correlated with weight change over time with an R value of 0.2413 (*p* < 0.001).

Compared to baseline, the first tertile lost −12.2% ± 5.7% (*p* < 0.0001), the second tertile lost −3.5% ± 1.4% (*p* < 0.0001), while the third tertile gained +2.8% ± 3.4% (*p* < 0.0001). The baseline and end of the study weight values for these tertiles were: (103.9 ± 19.8 kg vs. 91.2 ± 18.7 kg) for the first tertile, (103 ± 22 kg vs. 99.4 ± 21.3 kg) for the second tertile, and (102.4 ± 20.5 kg vs. 105.2 ± 21.2 kg) for the third tertile (Figure 1).

Compared to baseline, A1C decreased from 8.2% ± 1.4% to 7.3% ± 1.4% in the first tertile (*p* < 0.001), from 8.2% ± 1.6% to 7.6% ± 1.6% in the second tertile (*p* < 0.001), and from 8.0% ± 1.7% to 7.8% ± 1.6% in the third tertile (*p* = 0.4) (Table 2).

**TABLE 2:** Changes in metabolic parameters in patients who used GLP‐1 RA for 2.5 years in a real‐world clinical practice by tertile of percent weight loss.

	Tertile 1		Tertile 2		Tertile 3	
	Baseline	2.5 years	Baseline	2.5 years	Baseline	2.5 years
Weight (kg)	103.9 ± 2.14	91.2 ± 2**	103 ± 2.4	99.4 ± 2.3**	102.3 ± 2.2	105.2 ± 2.3
Body mass index (kg/m^2^)	34.8 ± 0.9	30.5 ± 0.8**	34.6 ± 0.9	33.4 ± 0.9**	34.4 ± 0.9	35.2 ± 0.9
A1 C (%)	8.3 ± 1.6	7.3 ± 1.6**	8.2 ± 1.6	7.6 ± 1.6**	8 ± 1.8	7.8 ± 1.8
Systolic blood pressure (mmHg)	128.4 ± 2.1	126 ± 1.9	129 ± 1.9	131.7 ± 2.05	129.3 ± 2.25	134.6 ± 2.03
Diastolic blood pressure (mmHg)	75.5 ± 1.3	74 ± 1.04	75.7 ± 1.4	75.9 ± 1.12	77.8 ± 1.12	77.8 ± 0.9
Total cholesterol (mg/dL)	141.6 ± 3.8	126.8 ± 4.05**	177.2 ± 6.5	161.9 ± 4.8*	154.2 ± 6.7	153 ± 6.8
LDL‐cholesterol (mg/dL)	69.7 ± 4.1	55.3 ± 3.5*	92.8 ± 6	81.7 ± 4.7*	85.4 ± 5.3	83.3 ± 5.5
HDL‐cholesterol (mg/dL)	46 ± 1.9	50.7 ± 2.2**	49.2 ± 2.2	53.3 ± 2.2*	45.3 ± 1.7	45 ± 1.8
Triglycerides (mg/dL)	183.9 ± 13.7	138 ± 8.1*	206.6 ± 22.5	158.3 ± 14.9**	160 ± 11.9	153.2 ± 12.07
Serum creatinine (mg/dL)	0.9 ± 05	0.9 ± 0.04	0.8 ± 0.03	0.9 ± 0.05	0.9 ± 0.05	1.0 ± 0.06*
Urinary microalbumin/creatinine ratio (μg/mg)	49.5 ± 22.2	26.5 ± 5.7	99.5 ± 44.06	256.06 ± 143.6	143.7 ± 77.8	216.2 ± 106.9
eGFR (mL/min/1.73 m^2^)	83.7 ± 3.5	84.2 ± 3.4	88.5 ± 3.4	84.2 ± 3.5*	86.1 ± 5.4	78.1 ± 4.2*
ALT (IU/L)	25.3 ± 3.2	20.3 ± 2	26.1 ± 2.6	24.5 ± 2.6	30.4 ± 3.9	28.1 ± 3.8
AST (IU/L)	22.3 ± 1.8	21.8 ± 1.8	20.5 ± 1.2	20.4 ± 1.9	27 ± 3.9	25.1 ± 3.2
Number of diabetes medications (*n*)	1.4 ± 0.13	1.3 ± 0.1	1.4 ± 0.1	1.4 ± 0.1	1.3 ± 0.1	1.1 ± 0.1
Number of antihypertensive medications (*n*)	1.3 ± 0.1	1.4 ± 0.1	1.5 ± 0.1	1.5 ± 0.1	1.3 ± 0.2	1.5 ± 0.2
Short acting insulin (units)	22.2 ± 4.3	15.2 ± 3.5*	14.4 ± 4.9	14.4 ± 4.8	19.5 ± 4	20.5 ± 3.8
Long acting insulin (units)	27.4 ± 4.5	19.3 ± 3.5*	19.2 ± 4	18.1 ± 3.8	30.6 ± 5	35.7 ± 5.8

*Note:* Data are given as mean ± SD.

* *p* < 0.05 compared to baseline.

** *p* < 0.001 compared to baseline.

Similarly, total serum cholesterol, HDL‐cholesterol, LDL‐cholesterol, and triglycerides improved significantly in the first and second tertiles but not in the third tertile (Table 2). In all tertiles, there were no significant changes from baseline in SBP, DBP, urinary microalbumin/creatinine ratio, liver enzymes, or utilization of antihyperglycemic and antihypertensive medications. Long‐ and short‐acting insulin doses were only reduced significantly in the first tertile (*p* < 0.05). Serum creatinine significantly increased in the third tertile (*p* = 0.02) and eGFR significantly decreased in the second (*p* = 0.02) and third tertiles (*p* = 0.01). All other changes in each tertile are shown in Table 2.

**FIGURE 2:**
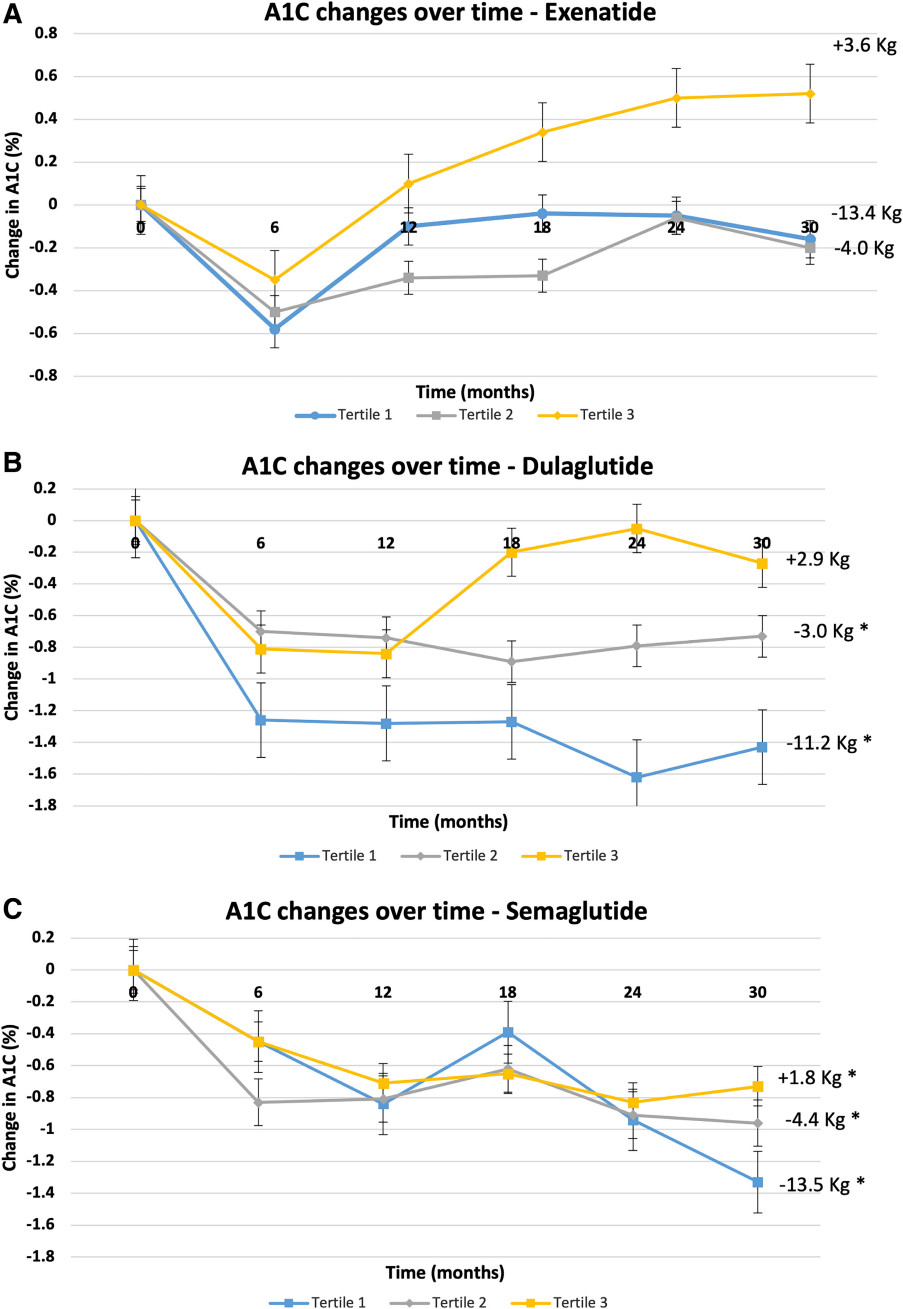
Change in A1C from baseline in each tertile of weight change over 2.5 years according to the used GLP‐1 RA. Data are given as mean ± SD. *p < 0.05 compared to baseline.

Changes in A1C and body weight over 2.5 years, by tertile, in each medication group is shown in Figure 2. Average change in body weight and A1C over 2.5 years by tertile in response to exenatide, dulaglutide, and semaglutide are shown in Tables 3 and 4, where the changes in response to dulaglutide and semaglutide are more consistent with the changes in the entire cohort.

**TABLE 3:** Average change in body weight over 2.5 years by tertile in response to exenatide, dulaglutide, and semaglutide.

Tertile group	Exenatide	Dulaglutide	Semaglutide
1st Tertile	−13.4 ± 7.7 kg (*p* < 0.0001)	−11.2 ± 6.5 kg (*p* < 0.0001)	−13.5 ± 5.6 kg (*p* < 0.0001)
2nd Tertile	−4 ± 2.04 kg (*p* < 0.0001)	−3 ± 1.36 kg (*p* < 0.0001)	−4.4 ± 2 kg (*p* < 0.0001)
3rd Tertile	+3.6 ± 5.06 kg (*p* < 0.001)	+2.9 ± 2.9 kg (*p* < 0.0001)	+1.8 ± 2.4 kg (*p* < 0.05)

*Note:* Data are given as mean ± SD. *p* < 0.05, considered statistically significant.

* *p* < 0.05 compared to baseline.

** *p* < 0.001 compared to baseline.

**TABLE 4:** Average change in A1C over 2.5 years by tertile in response to exenatide, dulaglutide, and semaglutide.

Tertile Group	Exenatide	Dulaglutide	Semaglutide
1st Tertile	−0.16% ± 2.17% (*p* = 0.6)	−1.4% ± 1.5% (*p* < 0.0001)	−1.3% ± 0.9% (*p* < 0.0001)
2nd Tertile	−0.18% ± 1.7% (*p* = 0.6)	−0.7% ± 1.2% (*p* < 0.05)	−0.9% ± 1.5% (*p* < 0.05)
3rd Tertile	+0.5% ± 1.7% (*p* = 0.1)	−0.3% ± 2.1% (*p* = 0.5)	−0.7% ± 1.5% (*p* < 0.05)

*Note:* Data are given as mean ± SD. *p* < 0.05, considered statistically significant.

* *p* < 0.05 compared to baseline.

** *p* < 0.001 compared to baseline.

## 4 Discussion

To our knowledge, this is the first longitudinal analysis in real‐world clinical practice to evaluate the long‐term relationship between changes in body weight and glycemic control in response to several GLP‐1 RAs. The findings of this analysis may shed some light on the complex relationships between weight loss, glycemic control, and cardiovascular risk factors in patients with T2D in response to such a popular category of antihyperglycemic medications, which currently have expanded indications beyond glycemic control. The important question to answer in this study is whether the changes in glycemic control and CV risk factors are predominately driven by weight loss over a long period of time versus an intrinsic effect of GLP‐1 RAs. The retrospective study design, despite its limitations, allowed us to collect information on patients who continued to use GLP‐1 RAs without interruption for 2.5 years and did not stop the medications because of side effects or lack of efficacy.

The study clearly showed that patients who lose and maintain weight loss for 2.5 years are those who get the maximal benefits of GLP‐1 RAs on A1C and CV risk factors; particularly dyslipidemia. On the contrary, patients who do not lose weight or instead gain weight, usually end up with minimal or no glycemic or lipid benefits. Furthermore, among patients who lost weight, the study showed that the magnitude of weight loss determines the degree of glycemic improvement.

Consistent with previous research, our study revealed a significant reduction in A1C levels, which aligns with the findings of other trials, such as SUSTAIN and REWIND, emphasizing a robust glucose‐lowering effect of GLP‐1 RAs in real‐world clinical practice [12, 13, 14]. Similarly, the effect of GLP‐1 RAs on weight loss in real‐world clinical practice was consistent with that observed by Marso et al. and Gerstein et al., with some difference in the magnitude of weight reduction [13, 14]. This discrepancy could be attributed to variations in the baseline demographics, BMI, or the differences in other antihyperglycemic medications used alongside GLP‐1 RAs.

In comparison to studies that mixed participants with variable weight responses into one single cohort, we divided them into tertiles based on their actual long‐term weight change. This allowed us to separately evaluate the impact of weight loss at different levels of clinical response to GLP‐1 RAs. The tertile that lost the least weight or gained weight showed the least glycemic benefit, whether in the total cohort or in response to any of the individual GLP‐1 RAs. Meanwhile, patients in the first tertile, who had the highest percentage of weight loss, reduced their insulin doses, while maintaining good glycemic control. The study adds to the existing literature by emphasizing the centrality of weight reduction in achieving good glycemic outcomes with GLP‐1 RAs.

Moreover, the study tried to explore the impact of the magnitude of weight change in response to GLP‐1 RAs on cardiovascular risk factors. Our findings showed improvements in lipid profile (total cholesterol, HDL, LDL, and triglycerides). These cardiovascular benefits are consistent with previous research and highlight the value of GLP‐1 RAs in addressing the broader metabolic dysfunctions associated with T2D [12, 13, 15, 16]. However, we did not see significant changes in BP in any of the 3 tertiles. Other long‐term weight loss studies showed that most of the benefit on blood pressure by weight reduction is seen in the first 18 months of weight loss, after which BP rebounds back even with the continuation of weight loss [17, 18]. Of note, no changes were seen in the number of antihypertensive medications during the observation period. Meanwhile, the progression of renal impairment continued in the tertile that gained weight. Other studies showed that maintenance of weight reduction is associated with improvement in kidney function in comparison to patients who regained weight [19].

From a clinical perspective, the study may direct the attention of healthcare providers to the magnitude of long‐term weight reduction as the main driver for glycemic and CV improvement. Considering that this group of medications significantly reduces lean muscle mass, which may be particularly risky in elderly patients, those who do not lose weight or rather gain weight while using GLP‐1 RAs, may not be suitable to continue using these medications [20]. On the contrary, other clinicians may argue the need to continue GLP‐1 RA use, considering their CV benefits. For this reason, it may be suggested that CV benefits evaluated in CV outcome trials should also re‐evaluate outcomes based on the magnitude of weight loss in post hoc analyses.

The study's strengths lie in its real‐world setting, analyzing comprehensive EHR from a specialized diabetes center, and exploring outcomes among three different GLP‐1 RAs. However, this study has several limitations. Despite the 2.5 years of follow‐up, a longer duration may be ideal. The study was conducted among patients treated in one center, so its generalizability is limited. The retrospective analysis prevented us from controlling other variables that might impact body weight or glycemic control. Furthermore, the study did not explore whether lifestyle modifications were incorporated alongside the use of GLP‐1 RAs. No information was collected about the nutrition intervention of this cohort. Although it was ideal to study the effects of tirzepatide in the same manner, the drug was used in the market for less than 2.5 years at the time this study was conducted. Investigators are currently collecting these data.

In conclusion, this study strongly suggests that long‐term improvement in glycemic control and lipid profile in response to GLP‐1 RAs is chiefly related to weight loss and minimally to any other intrinsic effects of GLP‐1 RA. This is particularly seen with long‐acting GLP‐1 RAs. Patients who lose little weight or gain weight on long‐term use of GLP‐1 RAs may not achieve better glycemic control. Future research should explore the longer‐term effects of GLP‐1 RAs and include more diverse populations to confirm and enhance the generalizability of our findings.

## Author Contributions

M.A. and S.D. have full access to all study data and take responsibility for data integrity and accuracy of data analysis. M.A. and S.D. designed the study, collected data, edited, reviewed, and prepared the manuscript for submission. O.H. designed and supervised the study, edited, and reviewed the manuscript. All authors approved the final version of the manuscript.

## Ethics Statement

The study was reviewed and approved by Joslin Diabetes Center's Committee on Human Studies (IRB approval ID: STUDY00000200) with a waiver of informed consent.

## Conflicts of Interest

O.H. receives research support from Eli Lilly and Novo Nordisk and serves on an advisory board for Abbott Nutrition. M.A. and S.D. have no disclosures relevant to this work.

## Data Availability

The data contained in this manuscript are held at the Joslin Diabetes Center clinical research center.
